# Supplementary material for: Polarized localization of kinesin-1 and RIC-7 drives axonal mitochondria anterograde transport
Source: bioRxiv. 2023 Jul 12:2023.07.12.548706. Preprint. [Version 1] doi: 10.1101/2023.07.12.548706 (PMC10369933; doi:10.1101/2023.07.12.548706)
Supplement: Supplement 8 [file NIHPP2023.07.12.548706v1-supplement-8.pdf]

bioRxiv preprint doi: https://doi.org/10.1101/2023.07.12.548706; this version posted July 12, 2023. The copyright holder for this preprint (which was not certified by peer review) is the author/funder, who has granted bioRxiv a license to display the preprint in perpetuity. It is made available under aCC-BY 4.0 International license.

Table S1. List of strains used in this study

Strain	Genotype
XE2194	*wpIs127[Pttx-3::mito::egfp; Pttx-3::mcherry::rab-3; Punc-122::gfp] X*
XE2257	*unc-116(rh24sb79) III; wpIs127[Pttx-3::mito::egfp; Pttx-3::mcherry::rab-3; Punc-122::gfp] X*
XE2322	*trak-1(wp84) I; wpIs127[Pttx-3::mito::egfp; Pttx-3::mcherry::rab-3; Punc-122::gfp] X*
XE3157	*trak-1(wy50182) I; wpIs127[Pttx-3::mito::egfp; Pttx-3::mcherry::rab-3; Punc-122::gfp] X*
XE2357	*miro-1(wp88) I; wpIs127[Pttx-3::mito::egfp; Pttx-3::mcherry::rab-3; Punc-122::gfp] X*
XE2447	*miro-3(wp94) I; miro-1(wp88) IV; miro-2(wp92) wpIs127[Pttx-3::mito::egfp; Pttx-3::mcherry::rab-3; Punc-122::gfp] X*
XE2366	*ric-7(n2657) V; wpIs127[Pttx-3::mito::egfp; Pttx-3::mcherry::rab-3; Punc-122::gfp] X*
XE3150	*miro-1(wp88) IV; ric-7(n2657) V; wpIs127[Pttx-3::mito::egfp; Pttx-3::mcherry::rab-3; Punc-122::gfp] X*
XE2651	*wpEx451[Pttx-3::mito::Dendra2::let-858 3'UTR+ Punc-122::gfp]*
XE2815	*miro-1(wp88) IV; wpEx451[Pttx-3::mito::Dendra2::let-858 3'UTR+ Punc-122::gfp]*
XE3158	*trak-1(wy50182) I; wpEx451[Pttx-3::mito::Dendra2::let-858 3'UTR+ Punc-122::gfp]*
XE2817	*wpEx449[Pitr-1pB::mito::TagRFP::let-858 3' UTR + Podr-1::GFP]*
XE2799	*ric-7(n2657) V; wpEx449[Pitr-1pB::mito::TagRFP::let-858 3' UTR + Podr-1::GFP]*
XE2798	*ric-7(wp127[ric-7::gfp11x7]) V; shyIs36[Pmig-13::gfp1-10+Podr-1::rfp] V wpEx483[Pitr-1pB::mito::TagRFP::let-858 3’ UTR + Podr-1::GFP]*

bioRxiv preprint doi: https://doi.org/10.1101/2023.07.12.548706; this version posted July 12, 2023. The copyright holder for this preprint (which was not certified by peer review) is the author/funder, who has granted bioRxiv a license to display the preprint in perpetuity. It is made available under aCC-BY 4.0 International license.

XE2929	*miro-1(wy50180) IV; ric-7(wp127[ric-7::GFP11x7]) V; shyIs36[Pmig-13::gfp1-10+Podr-1::rfp] V; wpEx483[Pitr-1pB::mito::TagRFP::let-858 3' UTR + Podr-1::GFP]*
XE2930	*mtx-2(wy50266) III; ric-7(wp127[ric-7::gfp11x7]) V; shyIs36[Pmig-13::gfp1-10+Podr-1::rfp] V; wpEx483[Pitr-1pB::mito::TagRFP::let-858 3' UTR + Podr-1::GFP]*
XE2931	*mtx-2(wy50266) III; miro-1(wy50180) IV; ric-7(wp127[ric-7::gfp11x7]) V; shyIs36[Pmig-13::gfp1-10+Podr-1::rfp] V; wpEx483[Pitr-1pB::mito::TagRFP::let-858 3' UTR + Podr-1::GFP]*
XE2917	*wpSi35[Pitr-1 pB::ric-7b::eGFP::let-858 3'UTR + Cbun-119(+)] I; wpEx449[Pitr-1pB::mito::TagRFP::let-858 3' UTR + Podr-1::GFP]*
XE2939	*wpSi40[Pmig-13::ric-7b::eGFP::let-858 3'UTR + Cbun-119(+)] I; ric-7(n2657) V; wpEx449[Pitr-1pB::mito::TagRFP::let-858 3' UTR + Podr-1::GFP]*
XE2940	*wpSi40[Pmig-13::ric-7b::eGFP::let-858 3'UTR + Cbun-119(+)] I; wpEx449[Pitr-1pB::mito::TagRFP::let-858 3' UTR + Podr-1::GFP]*
XE3041	*wpSi43[Pitr-1pB::ric-7b(1-470aa)::eGFP::let-858 3'UTR + Cbunc-119(+)] I; ric-7(n2657) V; wpEx449[Pitr-1pB::mito::TagRFP::let-858 3' UTR + Podr-1::GFP]*
XE2942	*wpSi36[Pmig-13::ric-7b(1-470aa)::eGFP::let-858 3'UTR + Cbun-119(+)] I; ric-7(n2657) V; wpEx449[Pitr-1pB::mito::TagRFP::let-858 3' UTR + Podr-1::GFP]*
XE2943	*wpSi36[Pmig-13::ric-7b(1-470aa)::eGFP::let-858 3'UTR + Cbun-119(+)] I wpEx449[Pitr-1pB::mito::TagRFP::let-858 3' UTR + Podr-1::GFP]*
XE2913	*wpSi28[Pitr-1 pB::ric-7b(1-99aa)::eGFP::let-858 3'UTR + Cbun-119(+)] I; ric-7(n2657) V; wpEx449[Pitr-1pB::mito::TagRFP::let-858 3' UTR + Podr-1::GFP]*
XE2933	*wpSi38[Pmig-13::ric-7b(1-99aa)::eGFP::let-858 3'UTR + Cbun-119(+)] I; ric-7(n2657) V; wpEx449[Pitr-1pB::mito::TagRFP::let-858 3' UTR + Podr-1::GFP]*
XE2934	*wpSi38[Pmig-13::ric-7b(1-99aa)::eGFP::let-858 3'UTR + Cbun-119(+)] I; wpEx449[Pitr-1pB::mito::TagRFP::let-858 3' UTR + Podr-1::GFP]*
XE2885	*wpSi30[Pitr-1 pB::ric7b(100-470aa)::eGFP::let-858 3'UTR + Cbun-119(+)] I ric-7(n2657) V wpEx449[Pitr-1pB::mito::TagRFP::let-858 3' UTR + Podr-1::GFP]*
XE2936	*wpSi39[Pmig-13::ric-7b(100-470aa)::eGFP::let-858 3'UTR + Cbun-119(+)] I; ric-7(n2657) V; wpEx449[Pitr-1pB::mito::TagRFP::let-858 3' UTR + Podr-1::GFP]*

bioRxiv preprint doi: https://doi.org/10.1101/2023.07.12.548706; this version posted July 12, 2023. The copyright holder for this preprint (which was not certified by peer review) is the author/funder, who has granted bioRxiv a license to display the preprint in perpetuity. It is made available under aCC-BY 4.0 International license.

XE2937	*wpSi39[Pmig-13::ric-7b(100-470aa)::eGFP::let-858 3'UTR + Cbun-119(+)] I; wpEx449[Pitr-1pB::mito::TagRFP::let-858 3' UTR + Podr-1::GFP]*
XE3045	*wpSi44[Pitr-1pB::ric-7b(471-709aa)::eGFP::let-858 3'UTR + Cbunc-119(+)] I; ric-7(n2657) V; wpEx449[Pitr-1pB::mito::TagRFP::let-858 3' UTR + Podr-1::GFP]*
XE2828	*wpSi22[rab-3p::ric-7a + Cbunc-119(+)] I; ric-7(n2657) V; wpIs127[Pttx-3::mito::egfp; Pttx-3::mcherry::rab-3; Punc-122::gfp] X*
XE2829	*wpSi23[rab-3p::human otulin + Cbunc-119(+)] I; ric-7(n2657) V; wpIs127[Pttx-3::mito::egfp; Pttx-3::mcherry::rab-3; Punc-122::gfp] X*
XE2830	*wpSi24[rab-3p::ric-7b 471-709aa+ Cbunc-119(+)] I; ric-7(n2657) V; wpIs127[Pttx-3::mito::egfp; Pttx-3::mcherry::rab-3; Punc-122::gfp] X*
XE2831	*wpSi25[rab-3p::human otulinL + Cbunc-119(+)] I; ric-7(n2657) V; wpIs127[Pttx-3::mito::egfp; Pttx-3::mcherry::rab-3; Punc-122::gfp] X*
XE3000	*unc-116(wp160[unc-116::3xgfp11]) III; shyIs36[Pmig-13::gfp1-10+Podr-1::rfp] V*
XE3005	*unc-116(wp160[unc-116::3xgfp11]) III; shyIs36[Pmig-13::gfp1-10+Podr-1::rfp] V; wpEx449[Pitr-1pB::mito::TagRFP::let-858 3' UTR + Podr-1::GFP]*
XE3134	*shyIs53[mig-13p::AtTir1+odr-1p::gfp] II; unc-116(shy92[unc-116::AID]) III; ric-7(wp127[ric-7::GFP11x7]) shyIs36[Pmig-13::gfp1-10+Podr-1::rfp] V; wpEx527[Pitr1-pB::mito::TagRFP::let858 3'UTR + Pmyo-2::mCherry]*

bioRxiv preprint doi: https://doi.org/10.1101/2023.07.12.548706; this version posted July 12, 2023. The copyright holder for this preprint (which was not certified by peer review) is the author/funder, who has granted bioRxiv a license to display the preprint in perpetuity. It is made available under aCC-BY 4.0 International license.

Table S2. List of plasmids used in this study:

pYW98	trak-1 5' sgRNA
pYW102	trak-1 3' sgRNA
pYW124	miro-1 5' sgRNA
pYW125	miro-1 3' sgRNA
pYW25	Pttx-3::mito::eGFP::let-858 3'UTR
pYW215	Pttx-3::mito::dendra2::let858 3'UTR
pYW217	Pitr-1pB::mito::TagRFP::let858 3'UTR
pYW264	Pitr-1pB::ric-7b::eGFP::let858 3'UTR
pYW278	Pmig-13::ric-7b::eGFP::let858 3'UTR
pYW274	Pitr-1pB::ric-7b(1-470aa)::eGFP::let858 3'UTR
pYW276	Pmig-13::ric-7b(1-470aa)::eGFP::let858 3'UTR
pYW262	Pitr-1pB::ric-7b(1-99aa)::eGFP::let858 3'UTR

bioRxiv preprint doi: https://doi.org/10.1101/2023.07.12.548706; this version posted July 12, 2023. The copyright holder for this preprint (which was not certified by peer review) is the author/funder, who has granted bioRxiv a license to display the preprint in perpetuity. It is made available under aCC-BY 4.0 International license.

pYW279	Pmig-13::ric-7b(1-99aa)::eGFP::let858 3'UTR
pYW261	Pitr-1pB::ric-7b(100-470aa)::eGFP::let858 3'UTR
pYW280	Pmig-13::ric-7b(100-470aa)::eGFP::let858 3'UTR
pYW309	Pitr-1pB::ric-7b(471-709aa)::eGFP::let858 3'UTR
pCD99	rab-3p::ric-7a
pCD102	rab-3p::human otulin
pCD101	rab-3p::ric-7b 471-709aa
pCD103	rab-3p::human otulinL

bioRxiv preprint doi: https://doi.org/10.1101/2023.07.12.548706; this version posted July 12, 2023. The copyright holder for this preprint (which was not certified by peer review) is the author/funder, who has granted bioRxiv a license to display the preprint in perpetuity. It is made available under aCC-BY 4.0 International license.

Table S3. List of gRNA sequences used in this study

sgRNA	Sequences
trak-1 5'	aaacatgttcactgccaaag
trak-1 3'	tcaaaaattaggtggagtgg
miro-1 5'	ggcgggattgataaatagaa
miro-1 3'	aacagagcgagagcaccaag
miro-2/3 5'	tttgttattgatggaacaat
miro-2/3 3'	caaaccagctagagctacta
7Xgfp11::miro-1	GATCAGAACGATTCGGACGT
ric-7:: 7Xgfp11	CCGGACGATGAATAATAACT
unc-116::3xgfp11<br>*unc-116::AID*	CCGTCAGCAGGAATGTCACA
MosSCI gRNA 1	GCGTCTTCGTACCTTTTTGG (Wang et al., 2016)
MosSCI gRNA 2	GTCGTTTTCTCTGAAGCGCA
